# Bioactivated Glucoraphanin Exerts a Potential Protective Effect in an In Vitro Model of Parkinson’s Disease: A Transcriptomic Study

**DOI:** 10.3390/ijms27188232

**Published:** 2026-09-16

**Authors:** Agnese Gugliandolo, Claudia Muscarà, Alessia Floramo, Veronica Argento, Luca Tagliavento, Francesco Meneguzzo, Daniele Perenzoni, Fulvio Mattivi, Emanuela Mazzon, Luigi Chiricosta

**Affiliations:** 1 IRCCS Centro Neurolesi “Bonino-Pulejo”, Via Provinciale Palermo, Contrada Casazza, 98124 Messina, Italy; 2 HyRes Srl, Via Salvator Rosa 18, 82100 Benevento, Italy; 3 CNR-IBE, Via Madonna del Piano 10, 50019 Sesto Fiorentino, Italy; 4 Department of Food Quality and Nutrition, Research and Innovation Centre, Fondazione Edmund Mach (FEM), Via E. Mach 1, 38098 San Michele all’Adige, Italy; 5 Center for Food Metabolomics and Bioactives Development (CSMETAB), 38098 Trento, Italy; 6 Department of Innovative Technologies in Medicine & Dentistry, University “G. d’Annunzio” Chieti-Pescara, Via Dei Vestini, 31, 66100 Chieti, Italy

**Keywords:** Parkinson’s disease, glucosinolates, isothiocyanates, glucoraphanin, myrosinase, sulforaphane, next-generation sequencing, transcriptome

## Abstract

Parkinson’s disease (PD) is a progressive neurodegenerative disorder characterized by both motor and non-motor symptoms. Oxidative stress is involved in disease pathogenesis, with the consequent damage to DNA. Nowadays, phytocompounds attract much interest for their capacity to modulate some of the pathogenic mechanisms of PD. Glucosinolates (GLs) and their derivatives, isothiocyanate (ITC), obtained by GLs hydrolysis by myrosinase (MYR), showed neuroprotective properties. In this study, we evaluated the protective effects of pre- and post-treatment with the GL glucoraphanin (GRA) bioactivated with MYR in an in vitro model of PD, namely differentiated SH-SY5Y cells treated with the toxin MPP^+^. With this aim, we performed transcriptomic analysis using next-generation sequencing (NGS). We observed that GRA + MYR pre-treatment modulated Biological Process (BP) of Gene Ontology (GO) and Reactome pathways associated with oxidative stress response and DNA repair mechanisms. In the post-treatment group, BP and pathways associated with DNA processes were also overrepresented in association with those correlated to oxidative stress. Indeed, some differentially expressed genes associated with oxidative stress response were upregulated, such as *SOD1*, whose protein levels also increased. The results of this exploratory study suggested that GRA + MYR may induce protective effects and, in particular, pre-treatment may modulate genes involved in DNA repair, while the post-treatment may exert a protective action against oxidative stress.

## 1. Introduction

Parkinson’s disease (PD) is a progressive neurodegenerative disorder characterized by a combination of motor symptoms, including bradykinesia, rigidity, tremor, and postural instability, as well as non-motor symptoms such as autonomic dysfunction, cognitive impairment, and sleep and mood disorders. From a neuropathological perspective, PD is associated with the selective loss of dopaminergic neurons in the substantia nigra and other brain regions, along with the accumulation of Lewy bodies, primarily composed of α-synuclein aggregates, which contribute to neuronal death [1]. Although standard therapy with L-DOPA compensates for dopamine deficiency, it does not halt disease progression and may, in some cases, contribute to increased oxidative stress through the production of reactive oxygen species (ROS) [2].

In this context, mitochondrial dysfunction and oxidative stress are considered central contributors to PD pathogenesis. In particular, impaired mitochondrial respiration can lead to excessive ROS production, which in turn induces oxidative damage to nuclear DNA. One proposed mechanism suggests that ROS generated by dysfunctional mitochondria may cause the accumulation of oxidative DNA lesions in nuclear DNA. This is particularly relevant for long neuron-specific genes, which are highly susceptible to transcriptional stress due to their size. DNA damage within these genes can stall RNA polymerase II progression, leading to impaired transcriptional fidelity, accumulation of mutations even in post-mitotic neurons, and ultimately progressive loss of neuronal integrity and cell death [3].

In recent years, there has been growing interest in the role of bioactive dietary compounds in modulating the pathogenic mechanisms of PD. Glucosinolates (GLs) and their isothiocyanate (ITC) derivatives, found in cruciferous vegetables, have shown promising neuroprotective properties [4]. GLs are phytochemicals naturally present in plants of the Cruciferae (Brassicaceae) family, as well as in *Moringa oleifera*, and are abundant in foods such as broccoli, cabbage, cauliflower, Brussels sprouts, kale, turnips, and wasabi. Under physiological conditions, GLs are stored separately from the enzyme myrosinase (MYR) within plant tissues. However, upon plant tissue disruption, such as cutting, chewing, or processing, MYR is released and catalyzes the hydrolysis of GLs into a variety of biologically active ITCs. The specific ITCs formed depend on the parent GL, and specifically, hydrolysis of glucoraphanin (GRA) leads to the formation of sulforaphane (SFN) [5].

GRA attracted considerable attention due to its ability to modulate redox-sensitive signaling pathways, particularly the Nrf2 pathway, a key regulator of cellular antioxidant defences. Preclinical studies demonstrated that GRA intake can increase the expression of Nrf2 and downstream antioxidant enzymes, such as heme oxygenase-1 (HO-1) and NQO1, thereby contributing to the protection of dopaminergic neurons in toxin-induced murine models of PD, including those based on MPTP [6].

Oxidative stress and neuroinflammation play central roles in the pathogenesis of neurodegenerative disorders. Bioactivated GRA demonstrated strong antioxidant and anti-inflammatory properties, which are largely responsible for its beneficial biological effects. Experimental evidence suggests that GRA may exert indirect neuroprotective effects through its enzymatic conversion to SFN, contributing to the reduction of dopaminergic toxicity and the maintenance of neuronal redox homeostasis [6]. Preclinical PD models showed that GRA-derived SFN resulted in a reduction of oxidative stress, one of the main factors involved in PD pathogenesis, as well as modulation of inflammatory processes and mitochondrial dysfunction [7,8]. SFN is also able to cross the blood–brain barrier (BBB) and exerts additional anti-inflammatory effects through inhibition of the MAPK and NF-κB signaling pathways [9].

In this study, we evaluated the effects of GRA bioactivated with MYR (GRA + MYR) as pre-treatment or post-treatment in an in vitro PD model. The aim of this study was to evaluate the potential protective effects of the GRA + MYR formulation in the PD model. With this aim, we evaluated the transcriptomic profile of differentiated SH-SY5Y cells treated with GRA + MYR as a pre-treatment or post-treatment in the 1-methyl-4-phenylpyridinium (MPP^+^)-induced PD model.

## 2. Results

### 2.1. Cell Viability Evaluation

**Figure 1:**
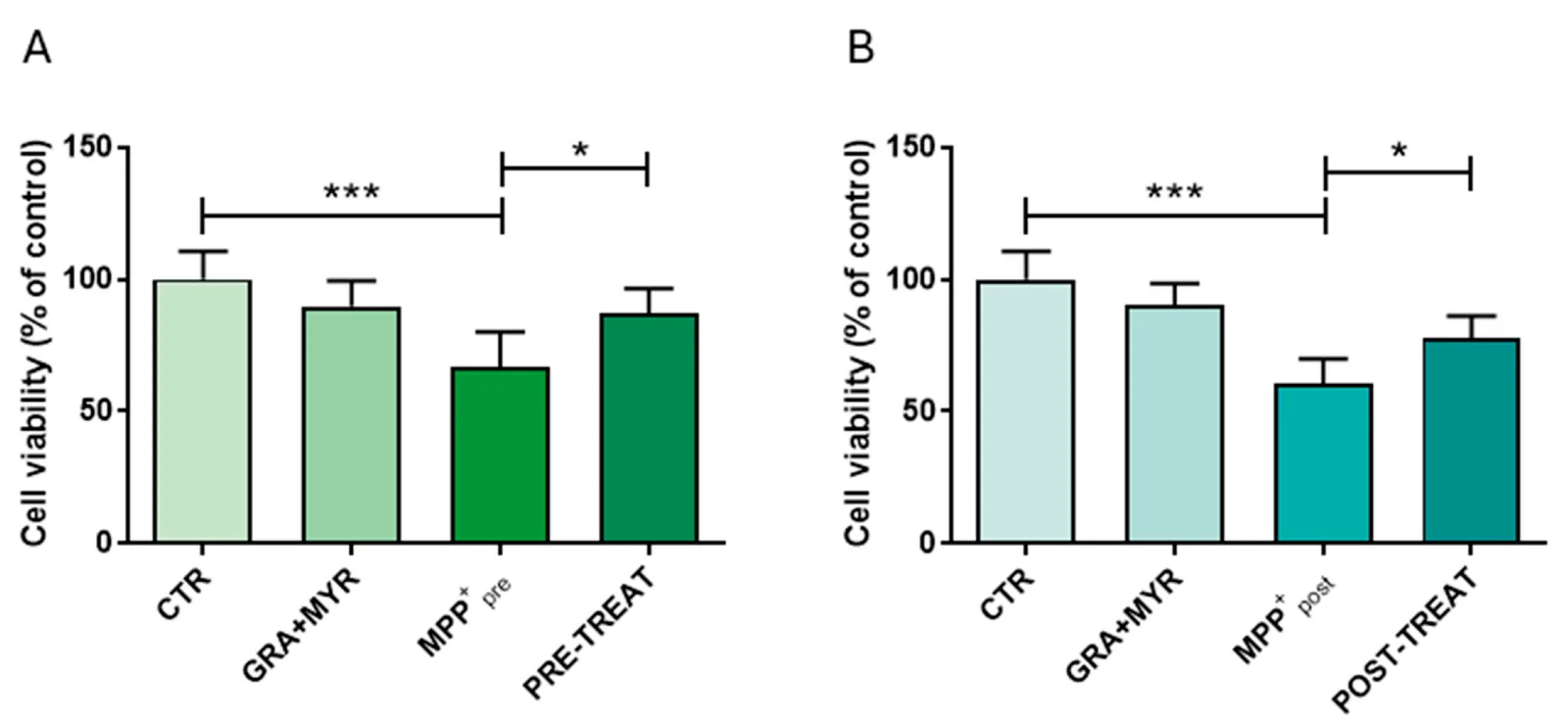
Evaluation of cell viability with GRA + MYR (**A**) pre-treatment and (**B**) post-treatment in an in vitro PD model. Both the pre- and post-treatment with GRA + MYR counteracted MPP^+^-induced reduction of cell viability. * *p* < 0.05; *** *p* < 0.001.

MPP^+^ treatment reduced cell viability compared to control cells (Figure 1). GRA + MYR was able to exert a protective action. A group was pre-treated with GRA + MYR for 24 h and then incubated with MPP^+^ for 48 h (PRE-TREAT). Another group was incubated with MPP^+^ for 48 h, followed by the 24 h post-treatment with GRA + MYR (POST-TREAT). Interestingly, both the pre- and the post-treatment were able to counteract the reduction of cell viability compared to MPP^+^ groups.

### 2.2. Transcriptomic Analysis of GRA + MYR Pre-Treatment in an In Vitro PD Model

**Figure 2:**
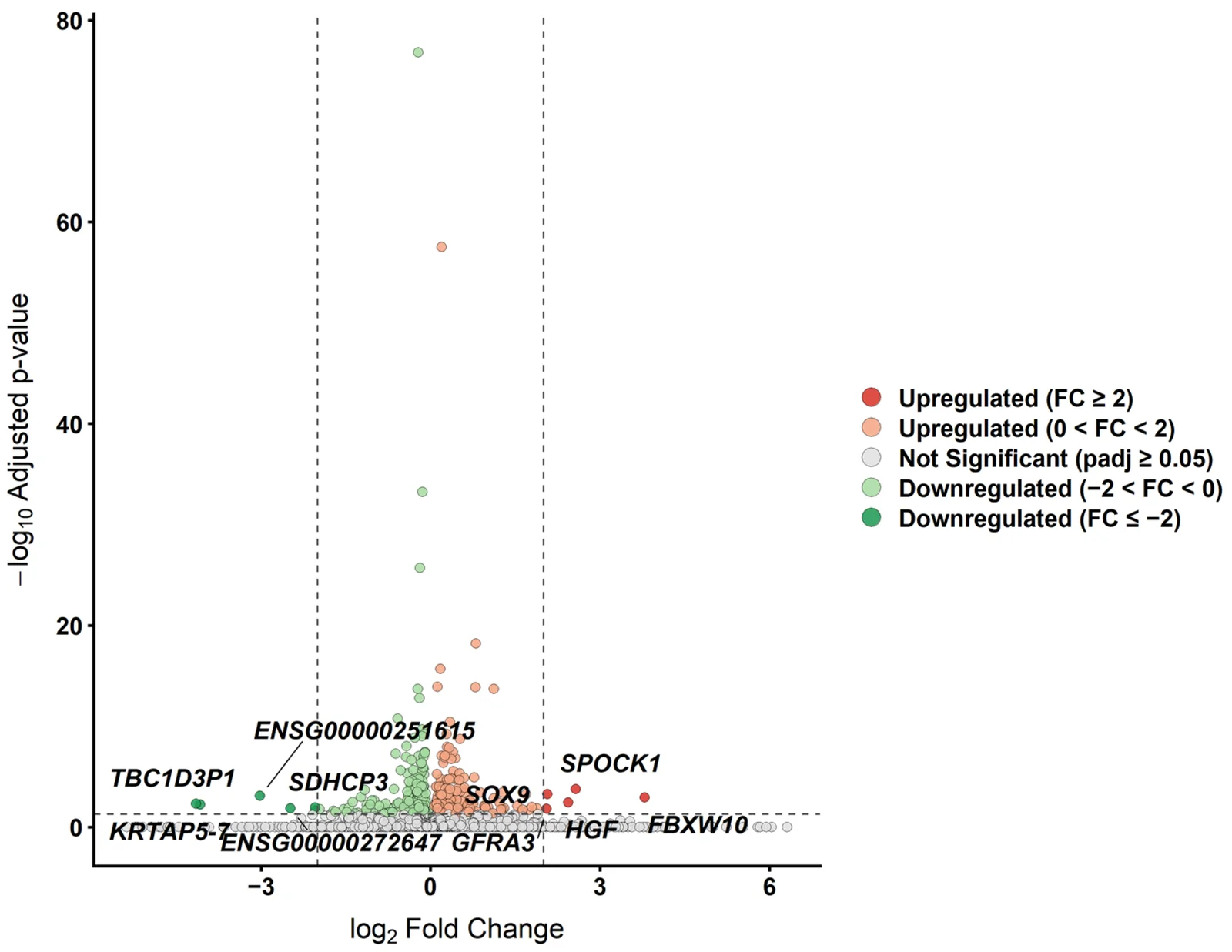
Volcano plots of the comparison MPP^+^_pre_ vs. PRE-TREAT. The overrepresented DEGs (more expressed in PRE-TREAT) are highlighted in red, dark red if log_2_ fold change (FC) was higher than 2. On the other hand, the downregulated DEGs (more expressed in MPP^+^_pre_) are depicted in green, dark green if log_2_ FC was lower than −2. Non-statistically significant genes are represented in gray. The fold change is computed as the log_2_(PRE-TREAT/MPP^+^_pre_). The score is computed as the log_10_(p-adjusted), and its threshold is set to the p-adjusted 0.05, highlighted by horizontal dash line. The gene names refer to the five most significant up- or downregulated DEGs with log_2_ FC ≥ 2 or ≤ -2, highlighted by vertical dash lines.

The comparative transcriptomic analysis of MPP^+^_pre_ vs. PRE-TREAT identified 591 differentially expressed genes (DEGs), of which 282 were upregulated and 309 downregulated in the PRE-TREAT group (Supplementary Table S1; Supplementary Figure S1). The distribution of DEGs is visible in the volcano plot in Figure 2. As it is possible to observe, the distribution of modulated genes is, in principle, symmetric between up- and downregulated DEGs. Even DEGs with level of log_2_ fold change (FC) higher than 2 or lower than −2 are comparable in amount.

#### 2.2.1. Gene Ontology (GO) Analysis for the Comparison MPP^+^_pre_ vs. PRE-TREAT

The analysis of GO revealed the overrepresentation of 263 different Biological Process (BP) of GO in the comparison MPP^+^_pre_ vs. PRE-TREAT (Supplementary Table S2) applying a log_2_ FC threshold of ≥2 or ≤−2. In accordance with the known bioactivated GRA effects, the GO analysis showed an involvement of response to oxidative stress (Table 1). Moreover, a BP related to DNA biosynthetic process was also overrepresented.

#### 2.2.2. Reactome Pathway Analysis for the Comparison MPP^+^_pre_ vs. PRE-TREAT

**Figure 3:**
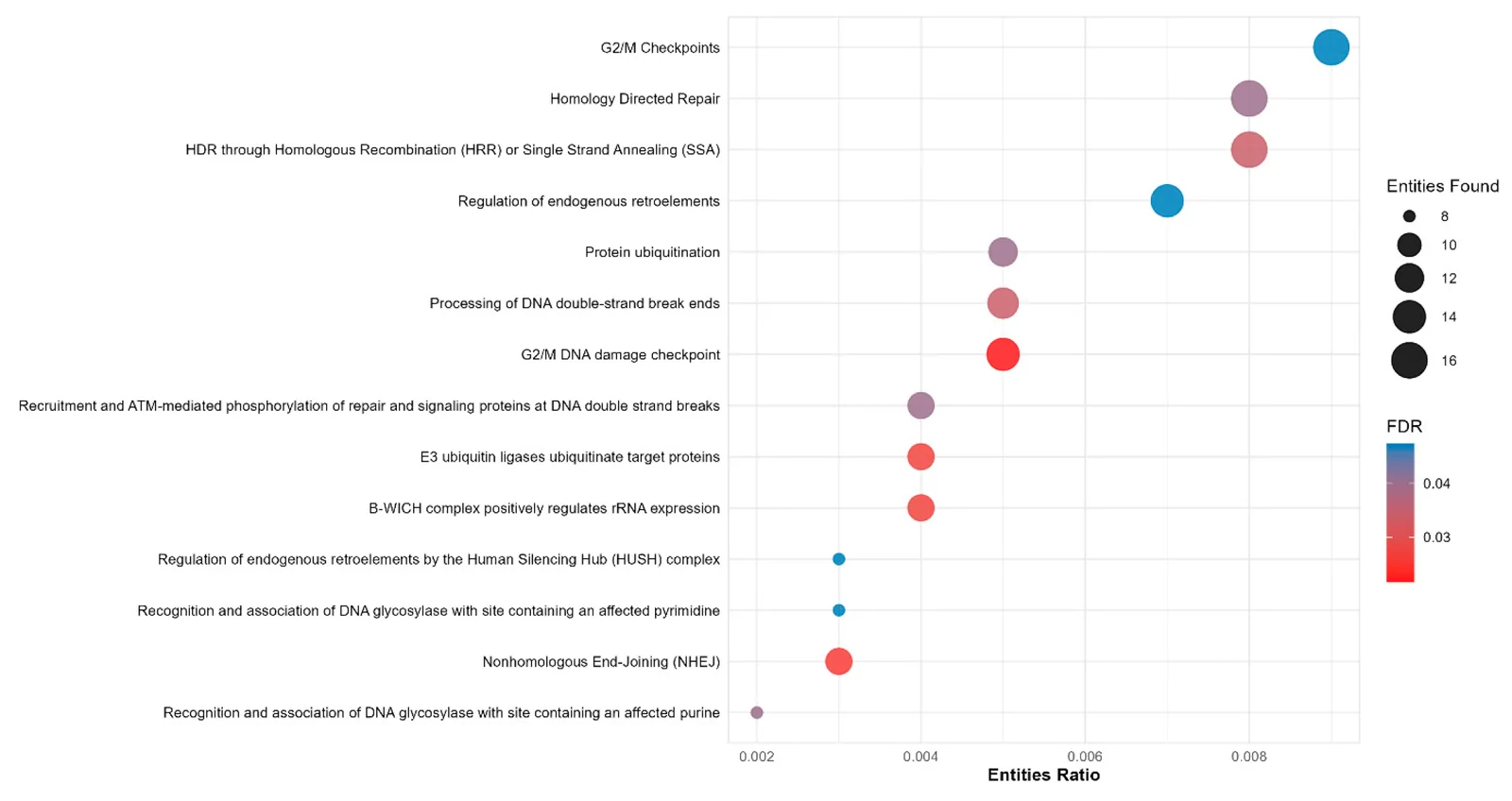
Overrepresented Reactome pathways in the comparison MPP^+^_pre_ vs. PRE-TREAT.

We better characterized the effects of GRA + MYR pre-treatment, analysing also Reactome pathways. At first, we evaluated the overrepresented pathways resulting from analysis of DEGs with log_2_ FC higher than 2 or lower than −2, and we found 14 pathways that were overrepresented (Supplementary Table S3), but they were not informative and not specific for this kind of study. Then, we analysed overrepresented pathways, eliminating the log_2_ FC cutoff in order to avoid omitting biologically relevant genes that exert key regulatory functions despite small changes in expression. This ensures the inclusion of essential pathway components that may act through fine-tuned or subtle expression changes. We found 14 overrepresented pathways (Figure 3). Interestingly, the majority were related to DNA repair mechanisms.

#### 2.2.3. Network and Cluster Analysis for the Comparison MPP^+^_pre_ vs. PRE-TREAT

To investigate the biological interactions among the DEGs, a network analysis was performed using the STRING database. We applied stringent criteria, retaining only “experimental” and “database” sources with a high-confidence interaction score (0.7).

**Figure 4:**
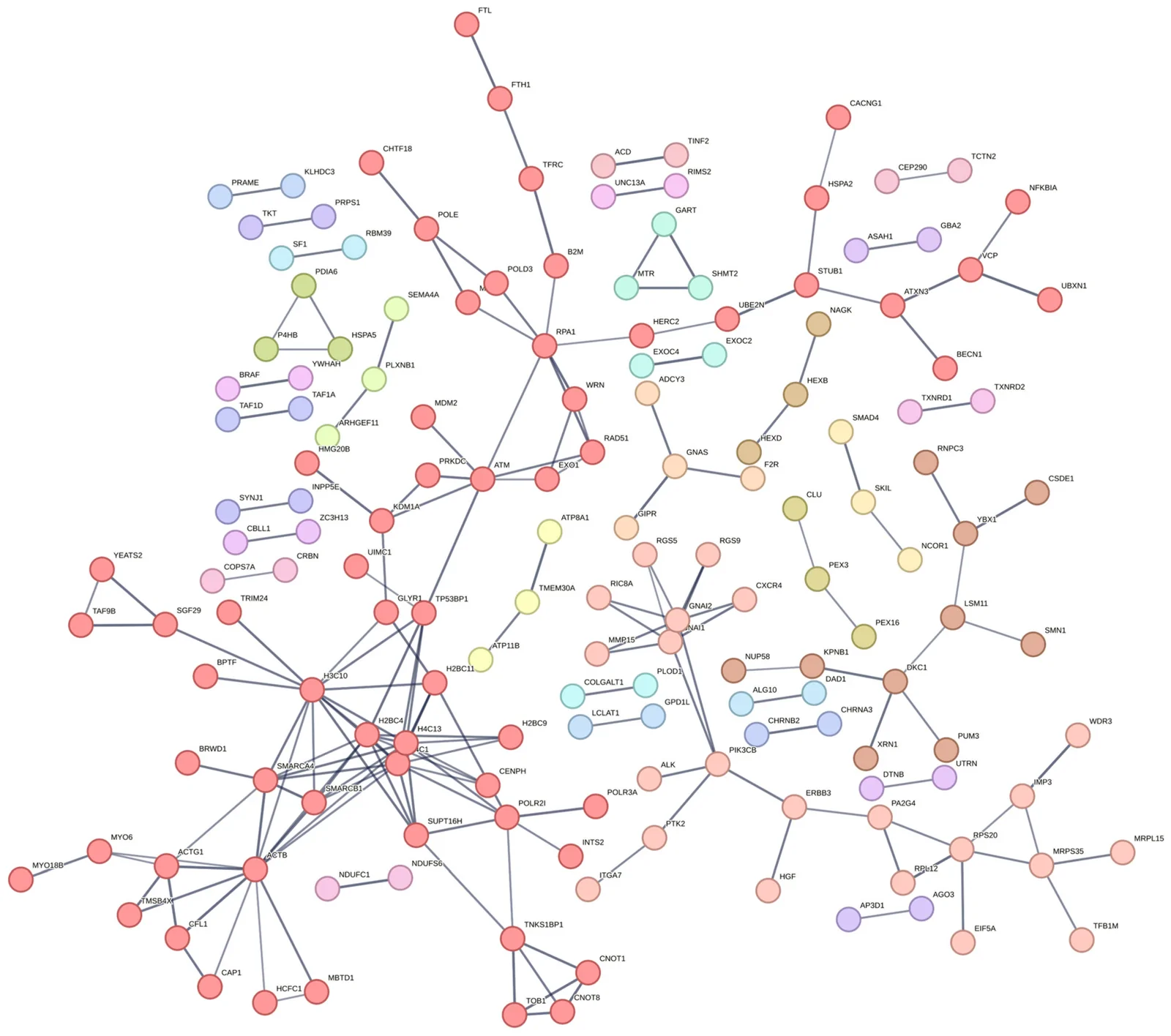
Network of MPP^+^_pre_ vs. PRE-TREAT comparison using k-means clustering. Each cluster of the network obtained using k-means clustering was highlighted with different colors. Red (largest) cluster is composed of 62 nodes and associated with “DNA Double-Strand Break Repair.”

For the MPP^+^_pre_ vs. PRE-TREAT comparison, the resulting network was composed of 32 disconnected components, as shown in Figure 4. In detail, the network counts 554 nodes, 188 edges with an average node degree of 0.679. K-means clustering (where k was set to the number of disconnected components) identified the largest cluster as being strictly related to “DNA Double-Strand Break Repair” (Supplementary Table S4). This cluster comprised 62 DEGs (34 upregulated, 28 downregulated in the PRE-TREAT group).

**Figure 5:**
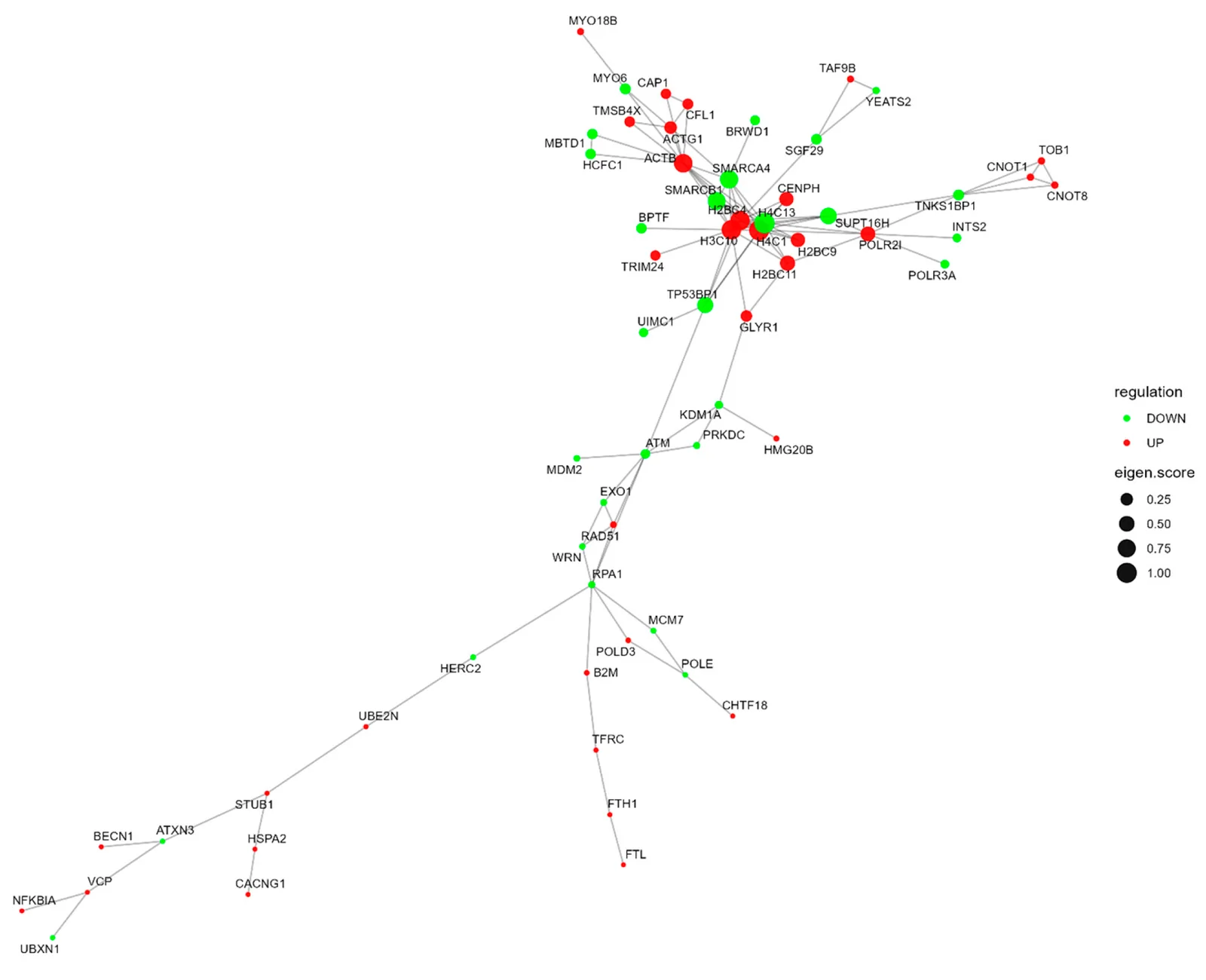
Role of DEGs in the largest cluster of the network of MPP^+^_pre_ vs. PRE-TREAT comparison. Each node of the subnetwork was associated with the regulation of the DEG in the comparison (green if downregulated, red if upregulated in the PRE-TREAT group). Additionally, the size of the node is representative of the strength of the DEG in the network (if bigger, it tends to be a hub node).

Since each node of the network is represented by a DEG in MPP^+^_pre_ vs. PRE-TREAT comparison, we inspected in detail in Figure 5 the up- or downregulation of the highlighted genes. Additionally, for each node, the eigenvalue score was used to represent the strength of the node as a hub in the network.

**Figure 6:**
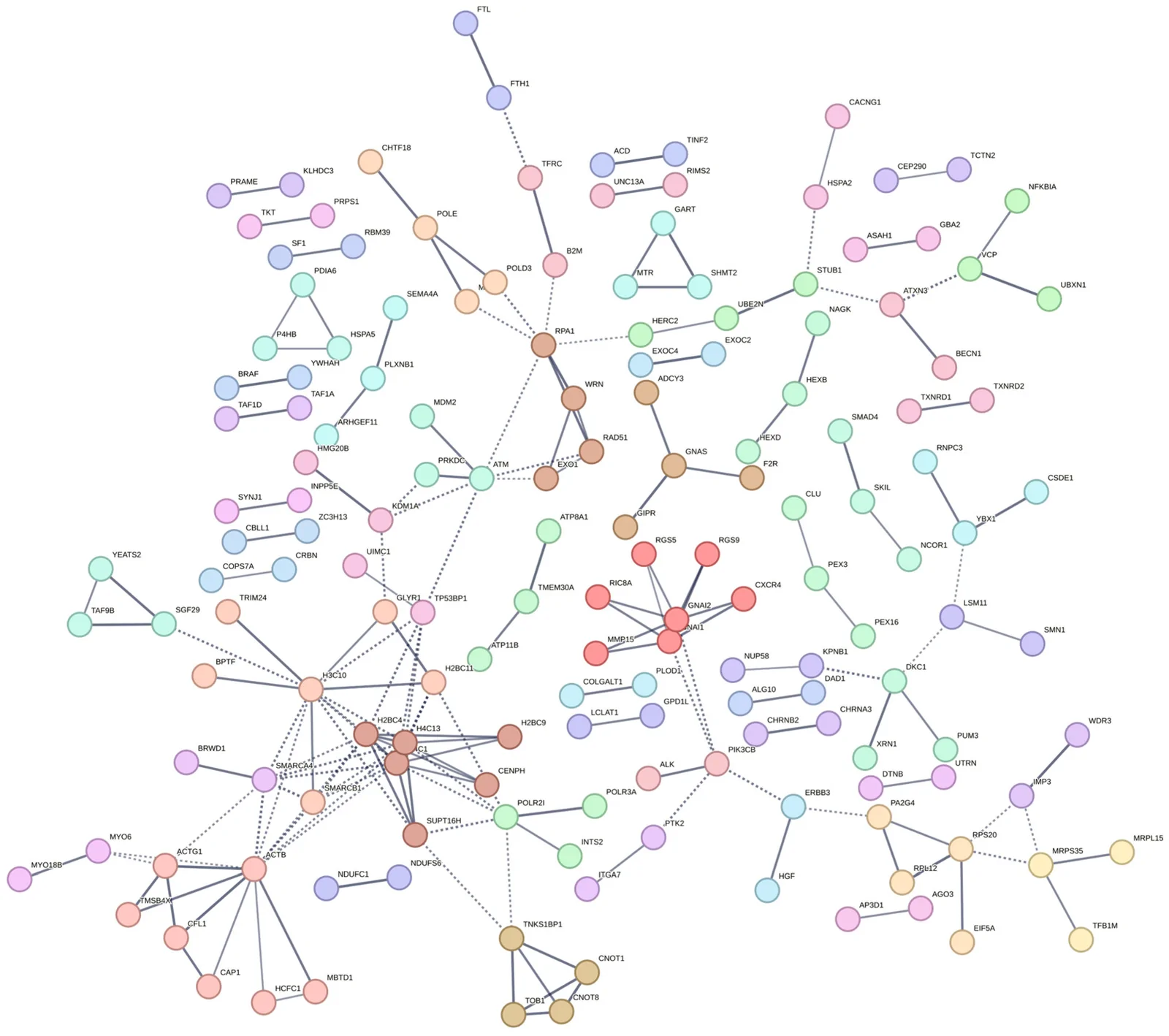
Network of MPP^+^_pre_ vs. PRE-TREAT comparison using MCL clustering. Each cluster of the network obtained using MCL clustering was highlighted with different colors. Dash lines indicated interactions between proteins of different clusters. Solid lines indicated interactions between proteins of the same cluster.

We also analyzed the STRING interaction networks using the Markov Cluster Algorithm (MCL) (Figure 6). Indeed, compared to k-means (which required setting k a priori and resulted in a very large, heterogeneous primary cluster), MCL successfully identified natural, densely connected modules based on network flow dynamics (Supplementary Table S5). Application of the MCL algorithm partitioned the network into discrete, highly interconnected functional clusters (Table 2). The larger, structurally connected modules captured general cellular responses, including signal transduction, but also highly specialized subclusters, whose genes were involved in DNA double-strand break repair and DNA replication-dependent/independent chromatin reorganization (Table 2). This modular partitioning suggested that GRA + MYR pre-treatment modulated DEGs involved in DNA processes.

### 2.3. Transcriptomic Analysis of GRA + MYR Post-Treatment in an In Vitro PD Model

**Figure 7:**
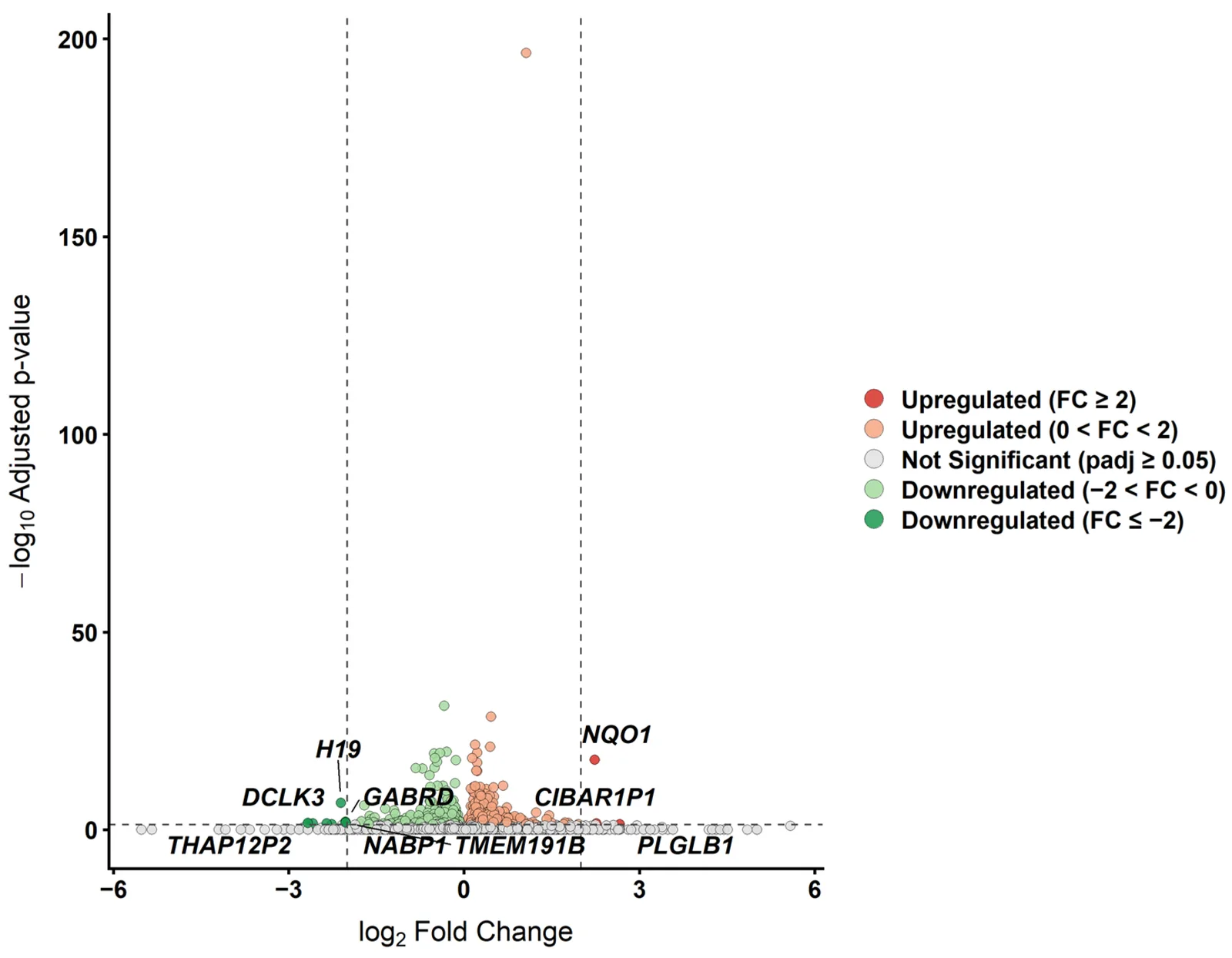
Volcano plots of the comparison MPP^+^_post_ vs. POST-TREAT. The overrepresented DEGs (more expressed in POST-TREAT) are highlighted red, dark red if FC was higher than 2. On the other hand, the downregulated DEGs (more expressed in MPP^+^_post_) are depicted in green, dark green if FC is lower than −2. Non-statistically significant genes are represented in gray. The fold change is computed as the log_2_(POST-TREAT/MPP^+^_post_). The score is computed as the log_10_(p-adjusted), and its threshold is set to the p-adjusted 0.05, highlighted by horizontal dash line. The gene names refer to the five most significant up- or downregulated DEGs with FC ≥ 2 or ≤−2, highlighted by vertical dash lines.

The comparison between MPP^+^_post_ vs. POST-TREAT revealed 799 DEGs, including 403 upregulated and 396 downregulated genes in the POST-TREAT group (Supplementary Table S6; Supplementary Figure S2). The distribution of DEGs is visible in Figure 7, and even in this case, the amount of up- and downregulated DEGs are quite similar both under and over the FC threshold.

#### 2.3.1. GO Analysis for the Comparison MPP^+^_post_ vs. POST-TREAT

The analysis of GO revealed the overrepresentation of 11 different BP of GO in the comparison MPP^+^_post_ vs. POST-TREAT, applying a log_2_ FC higher than 2 or lower than −2. (Table 3). Also in this comparison, processes related to oxidative stress were significantly overrepresented, suggesting a potential antioxidant effect of GRA + MYR in POST-TREAT.

#### 2.3.2. Reactome Pathway Analysis for the Comparison MPP^+^_post_ vs. POST-TREAT

**Figure 8:**
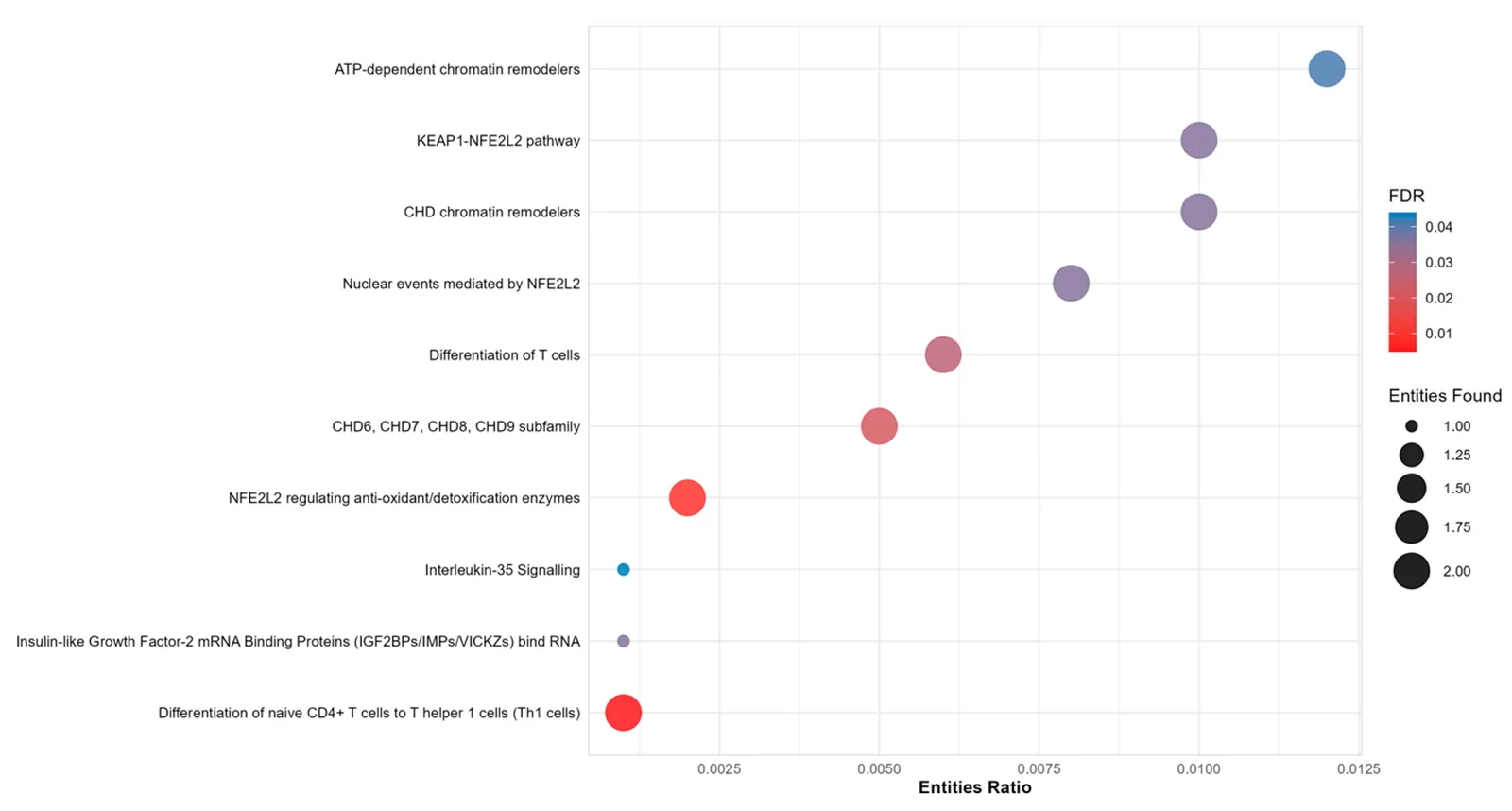
Overrepresented Reactome pathways in the comparison MPP^+^_post_ vs. POST-TREAT using DEGs with log_2_ FC higher than 2 or lower than −2.

**Figure 9:**
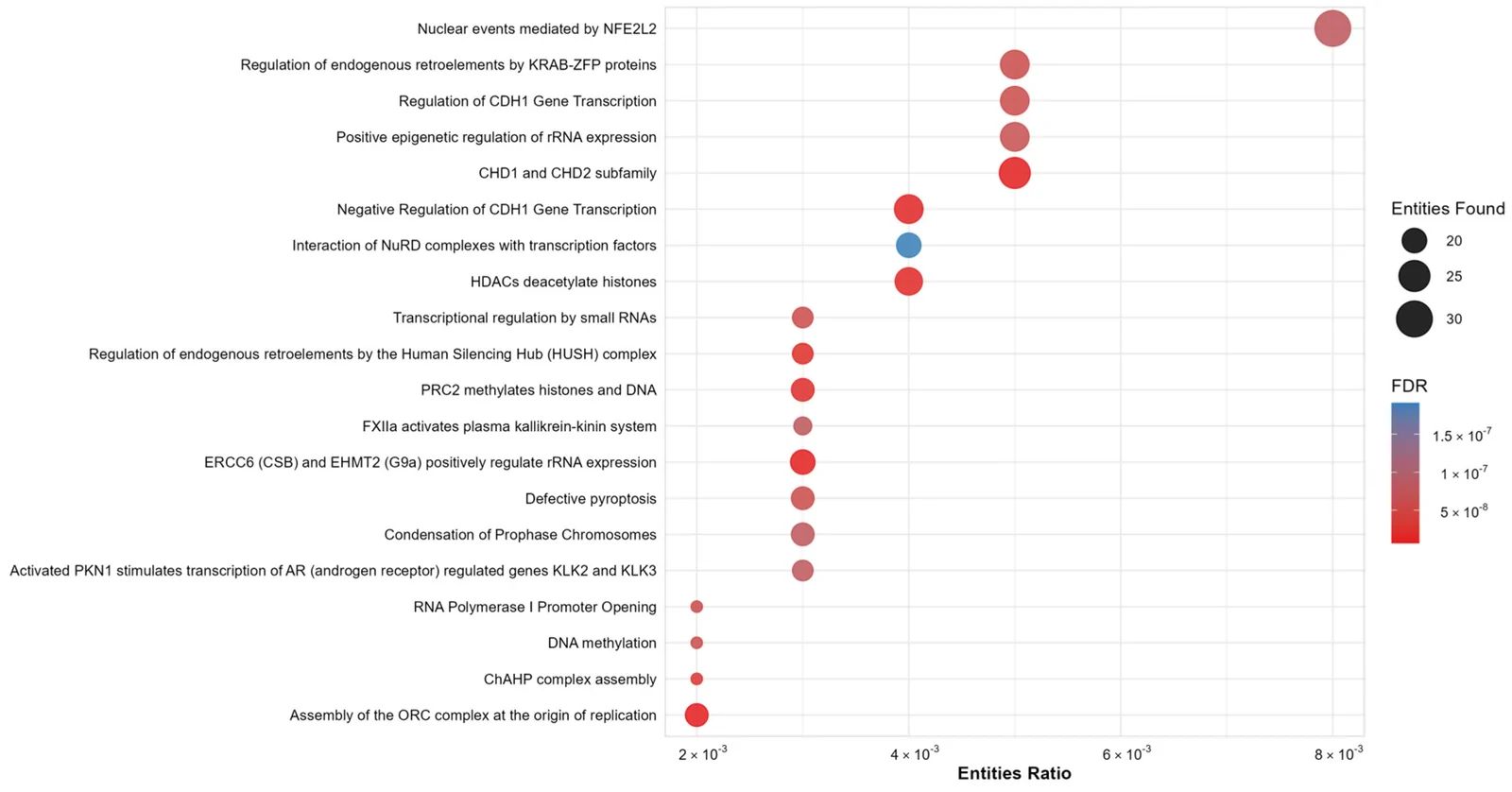
The 20 overrepresented Reactome pathways in the comparison MPP^+^_post_ vs. POST-TREAT.

We found 10 overrepresented pathways in the MPP^+^_post_ vs. POST-TREAT comparison, applying the cutoff of log_2_ FC higher than 2 or lower than −2. Overrepresented pathways indicated an effect of GRA + MYR on oxidative stress and chromatin remodelling (Figure 8). We repeated the analysis, eliminating the FC cutoff (Supplementary Table S7). The 20 most significant pathways are available in Figure 9. Interestingly, most of the pathways were related to DNA and RNA processes, such as transcription. However, the pathway “Nuclear events mediated by NFE2L2” was also overrepresented.

#### 2.3.3. Network and Cluster Analysis for the Comparison MPP^+^_post_ vs. POST-TREAT

**Figure 10:**
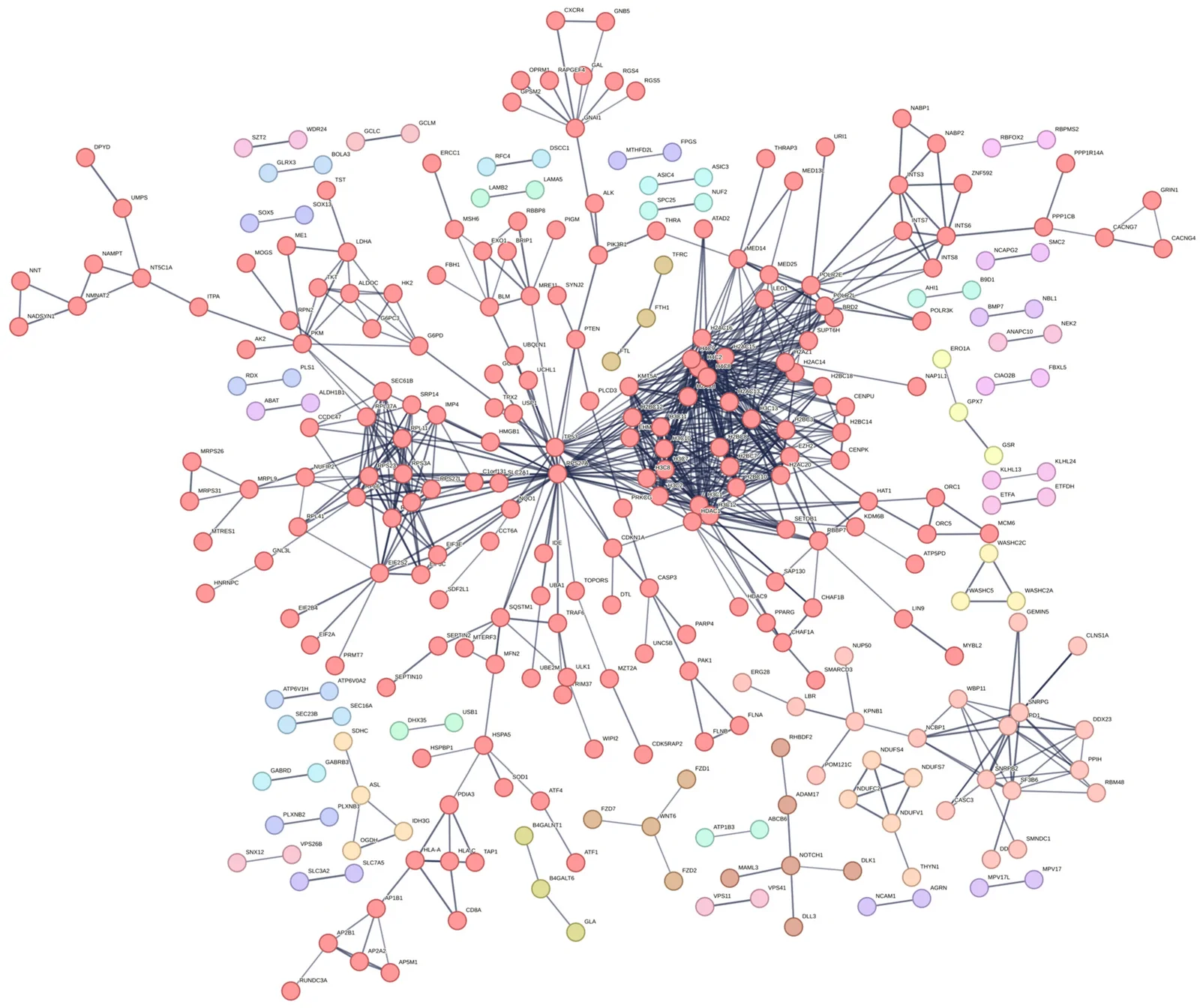
Network of MPP^+^_post_ vs. POST-TREAT comparison. Each cluster of the network obtained using k-means clustering was highlighted with different colors. Red (largest cluster) is composed of 194 nodes without unique classification.

In the MPP^+^_post_ vs. POST-TREAT analysis, the network, represented in Figure 10, showed 40 disconnected components. Specifically, 761 nodes and 769 edges were, respectively, observed, and the average node degree was 2.02. The initial k-means clustering produced a large cluster of 194 genes (98 upregulated, 96 downregulated in POST-TREAT group) without a specific functional enrichment (Supplementary Table S8).

**Figure 11:**
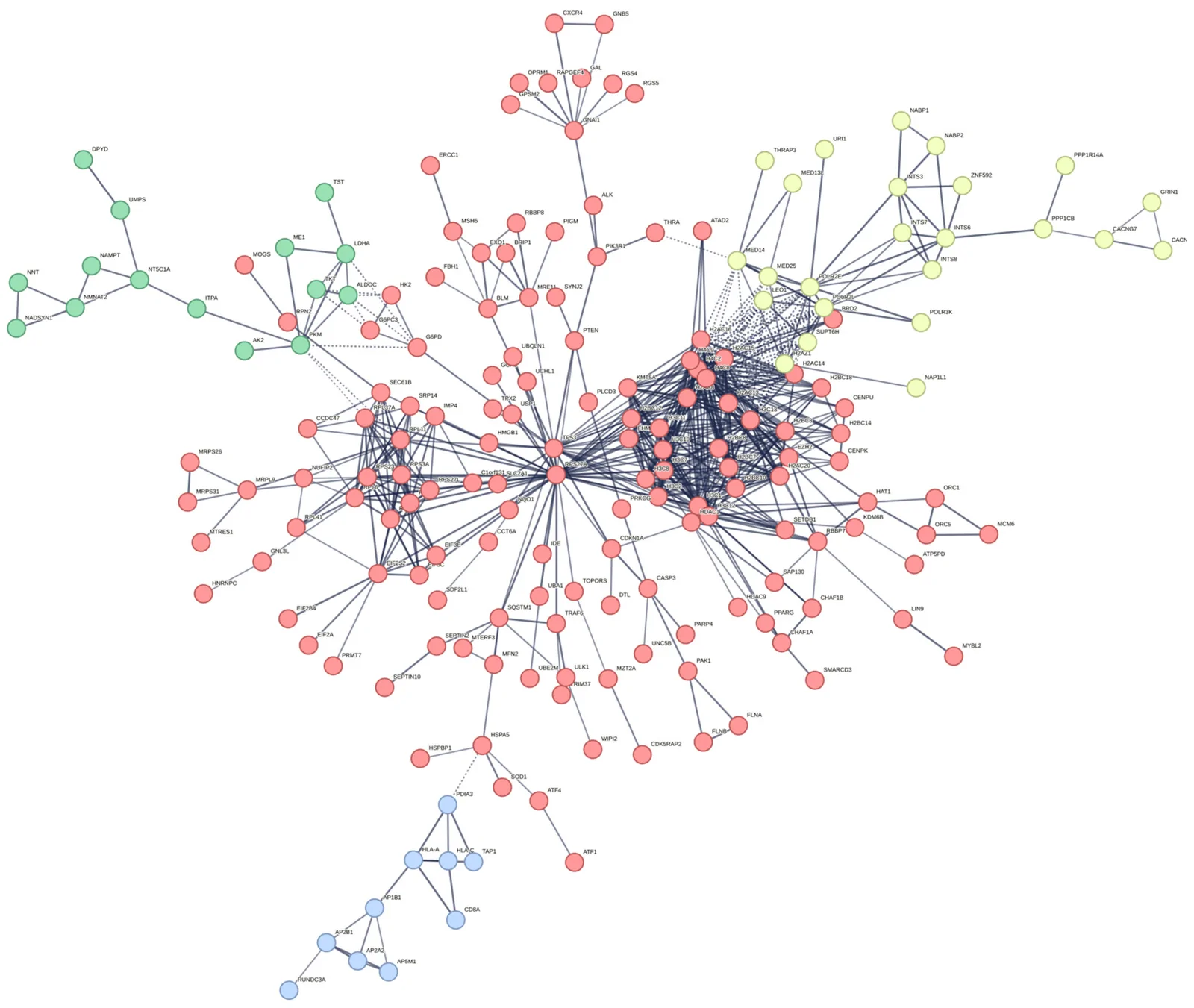
Largest cluster of the network of MPP^+^_post_ vs. POST-TREAT comparison. Each cluster of the subnetwork obtained using k-means clustering was highlighted with different colors. The identified clusters were “Chromatin organization” (red), “Regulation of transcription elongation from RNA polymerase II promoter/Integrator complex” (yellow), “Nicotinate and nicotinamide metabolism” (green), and “Antigen processing and presentation/Nef-mediated down-modulation of cell surface receptors” (blue). Dash lines indicated interactions between proteins of different clusters. Solid lines indicated interactions between proteins of the same cluster.

To achieve higher resolution, a secondary clustering step was performed (Supplementary Table S9). By setting the algorithm to identify a minimum of four subclusters, we successfully characterized in Figure 11 the functional modules “Chromatin organization” (145 genes, the largest module, 79 upregulated and 66 downregulated in POST-TREAT group), “Regulation of transcription elongation from RNA polymerase II promoter/Integrator complex” (24 genes, 9 upregulated and 15 downregulated in POST-TREAT group), “Nicotinate and nicotinamide metabolism” (15 genes, 6 upregulated and 9 downregulated in POST-TREAT group), and “Antigen processing and presentation/Nef-mediated down-modulation of cell surface receptors” (10 genes, 4 upregulated and 6 downregulated in POST-TREAT group).

**Figure 12:**
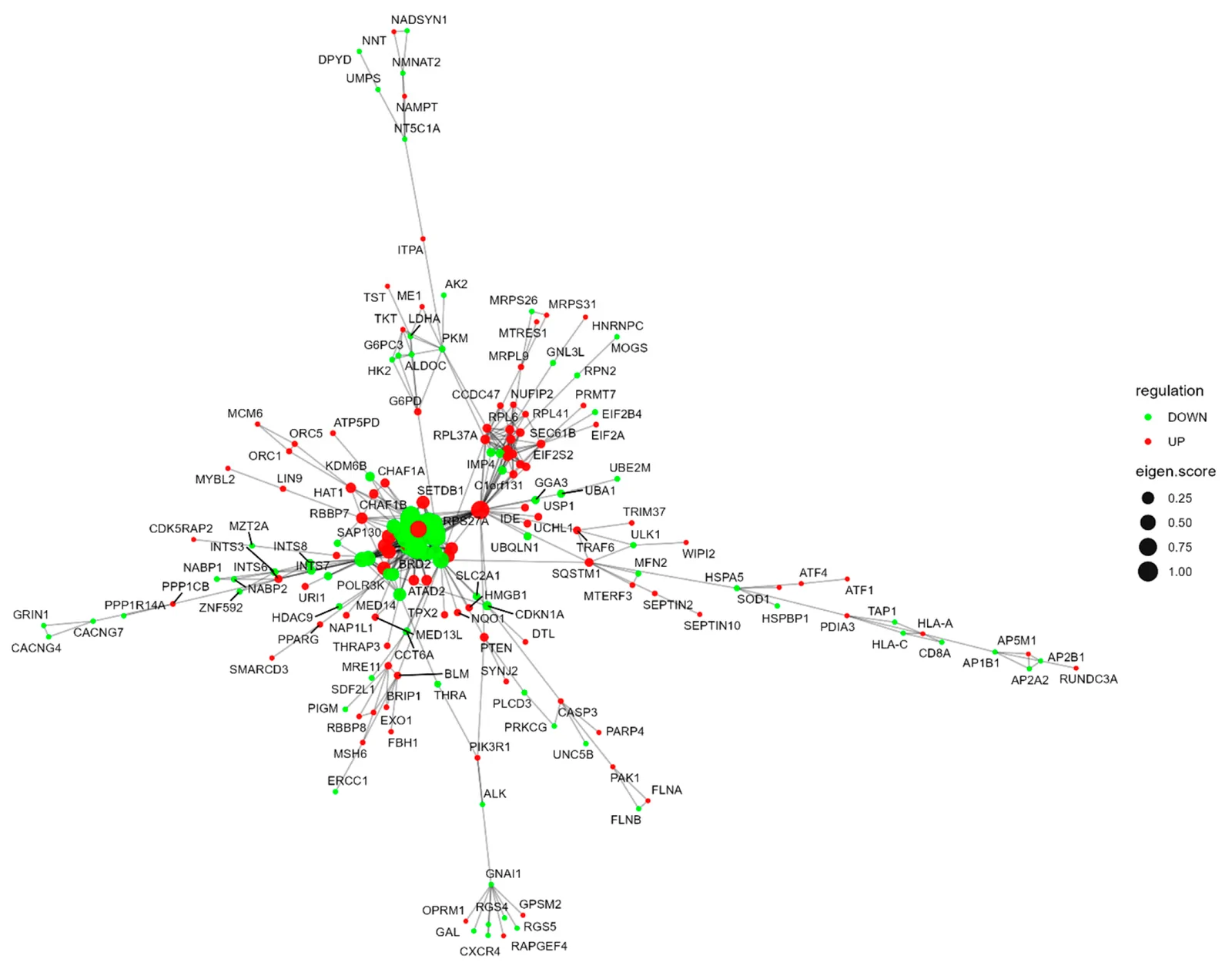
Role of DEGs in the largest cluster of the network of MPP^+^_post_ vs. POST-TREAT comparison. Each node of the subnetwork was associated with the regulation of the DEG in the comparison (green if downregulated, red if upregulated). Additionally, the size of the node is representative of the strength of the DEG in the network (if bigger, it tends to be a hub node).

Even for the MPP^+^_post_ vs. POST-TREAT comparison, in Figure 12, each node is represented with its positive or negative regulation, along with eigenvalue score.

**Figure 13:**
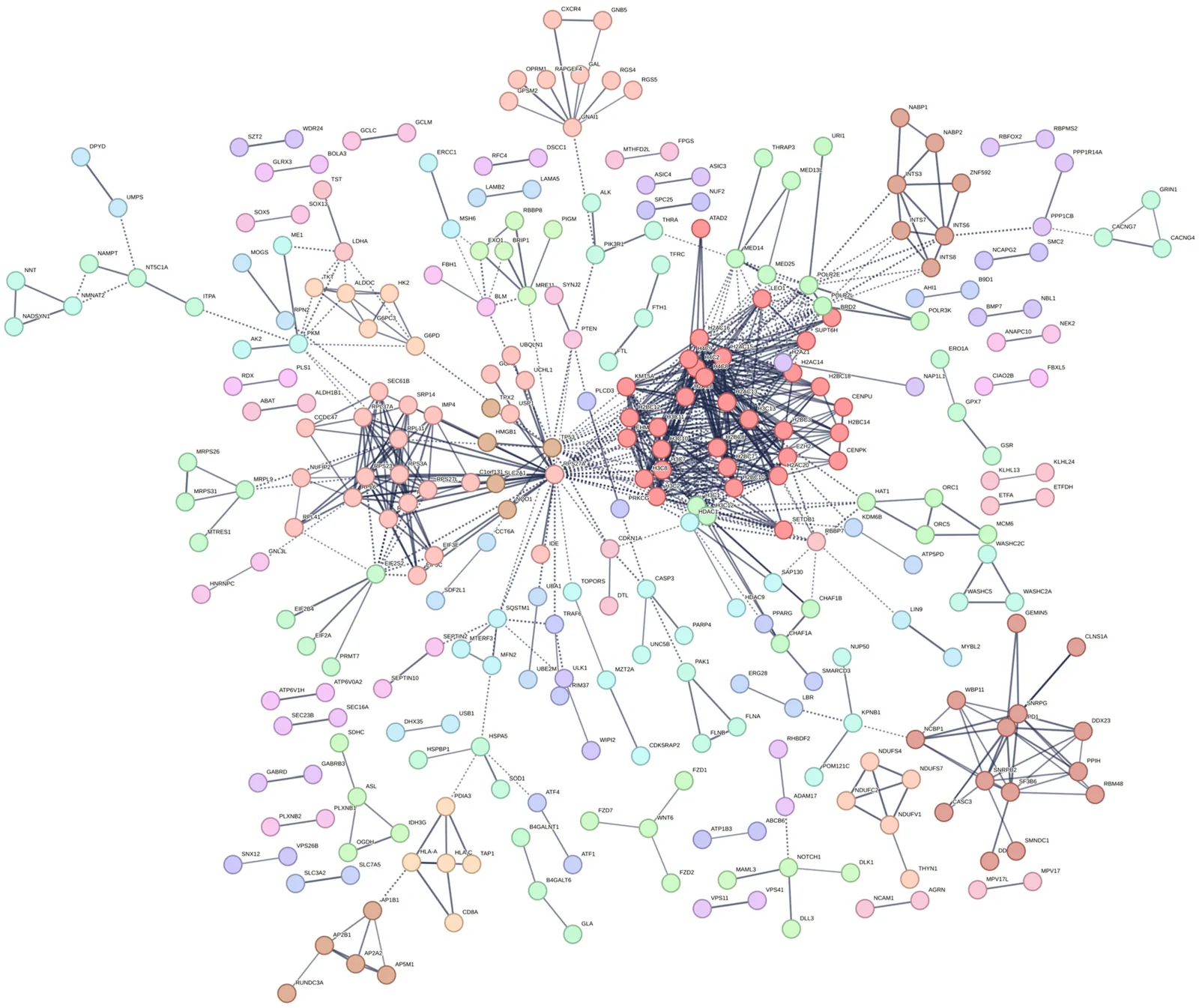
Network of MPP^+^_post_ vs. POST-TREAT comparison using MCL clustering. Each cluster of the network obtained using MCL clustering was highlighted with different colors. Dash lines indicated interactions between proteins of different clusters. Solid lines indicated interactions between proteins of the same cluster.

We performed even MCL clustering analysis for the comparison MPP^+^_post_ vs. POST-TREAT (Figure 13; Supplementary Table S10). Also in this case, the major clustering refers to chromatin organization (Structural constituent of chromatin Nucleosome: *H3C13*, *H4C8*, *H4C2*, *H4C9*, *H3C11*, *H3C7*, *H3C8*, *H2AC8*, *H3C10*, *H2BC12*, *H3C2*, *KMT5A*, *EHMT2*, *SETDB1*, *H2BC7*, *H2BC8*, *H2BC10*, *H2AC15*, *H2AC16*, *H2AC13*, *CENPU*, *CENPK*, *ATAD2*, *LEO1*, *SUPT6H*, *EZH2*, *H2AC14*, *H2BC3*, *H2AC20*, *H2BC18*, *H2BC14*, and *BRD2*).

### 2.4. Western Blot of SOD1

**Figure 14:**
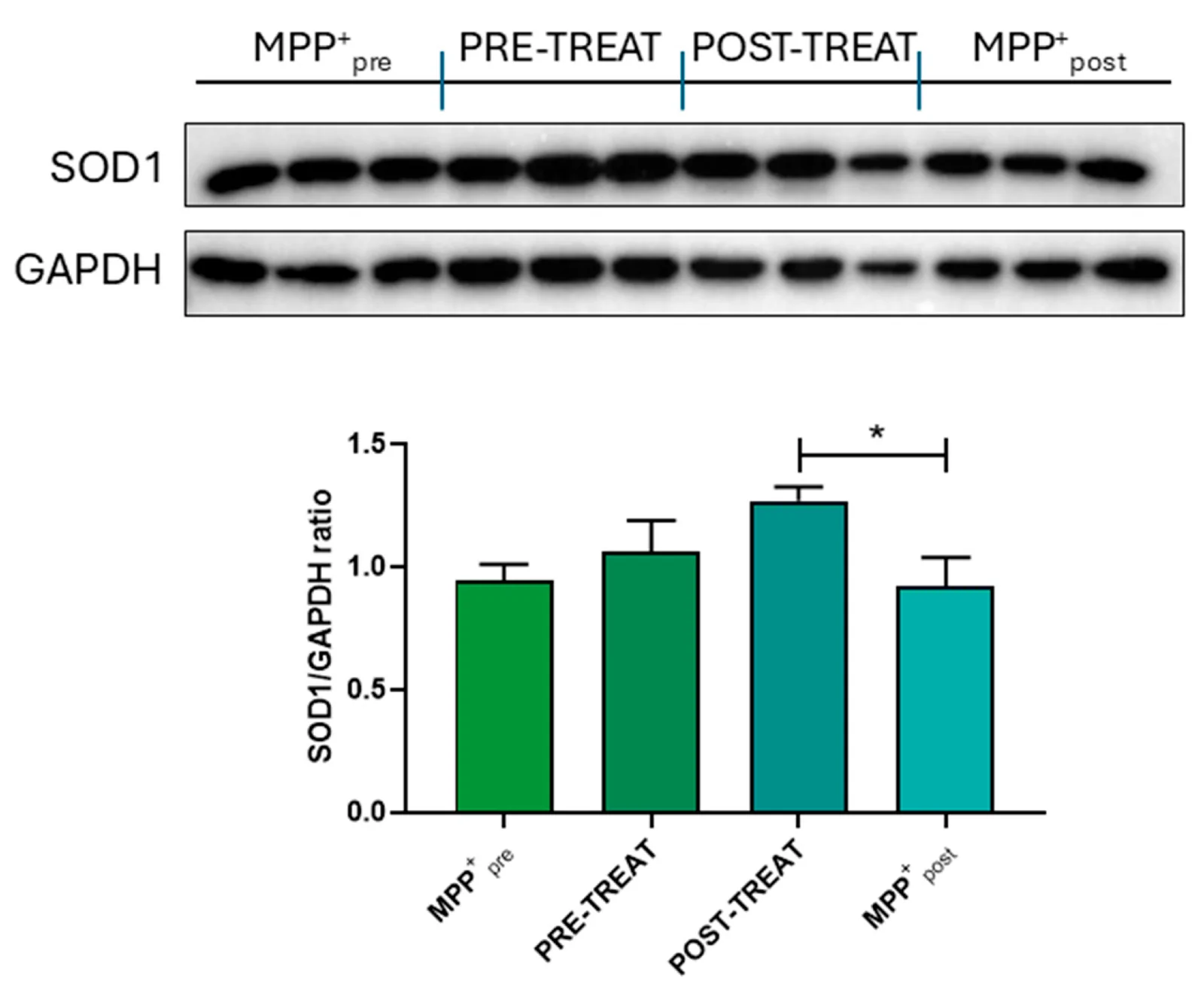
Western blot analysis of SOD1. A significant increase in SOD1 levels was observed in the MPP^+^_post_ vs. POST-TREAT comparison. * *p* < 0.05.

In order to confirm transcriptomic data and the antioxidant effect of GRA + MYR, we performed Western blot analysis of SOD1. According to transcriptomic data, a significant increase in the levels of SOD1 was observed in POST-TREAT compared to MPP^+^_post_ (Figure 14). Interestingly, we observed the same trend also in the PRE-TREAT group, but it was not statistically significant, in line with transcriptomic data.

## 3. Discussion

Phytocompounds such as GLs and ITCs are arising as promising agents for the treatment of neurodegenerative diseases. SFN, that is the ITC produced by GRA bioactivation, received increasing attention as a potential neuroprotective compound in different neurodegenerative diseases [9,10], also thanks to its bioavailability and the capacity to cross the BBB [11].

In this study, we produced an extract of seed of Tuscan black kale rich in GRA and an extract of seed of *Sinapis alba* with MYR activity through hydrodynamic cavitation. We produced these extracts to reduce to practice the patent EP2908850B1. Furthermore, given that these formulations were intended for administration in a clinical trial (NCT07360977), high standards of activity and purity were required. To our knowledge, no commercially available MYR preparations meet these food/clinical-grade criteria.

In this study, we observed that GRA + MYR was able to counteract the loss of cell viability induced by MPP^+^. In particular, we chose a sublethal dose of MPP^+^ that induces about 30% of loss of cell viability compared to control. This reduction of cell viability represents an optimal window to study molecular mechanisms and the neuroprotective effects of potential protective compounds.

In a previous study, our group demonstrated that bioactivated GRA protected neurons thanks to anti-apoptotic, anti-oxidative, and anti-inflammatory actions in an in vivo PD model [12]. In line with these results, a study indicated that dietary intake of GRA prevented the reduction of dopamine transporter in the mouse striatum after MPTP-repeated administration [6]. Also, the treatment with SFN showed protective effects mainly mediated by its antioxidant and anti-inflammatory actions. Indeed, a study found that the pre-treatment with SFN reduced 6-OHDA-induced apoptosis, while increasing HO-1 expression, Nrf2 translocation into the nucleus, and PI3K/Akt activation [13]. SFN pre-treatment can also prevent ER stress induced by 6-OHDA due to the activation of Nrf2 [14]. Moreover, SFN had the ability to enhance glutathione levels and its dependent enzymes and to modulate neuronal survival pathways, such as ERK1/2, in the brain of mice [15]. SFN also inhibited NLRP3 inflammasome activation by inducing mitochondrial autophagy and mitigating CBS-H2S axis damage in PD in vitro and in vivo models [16].

However, some studies also evidenced a reduced DNA fragmentation after treatment with SFN. Specifically, SFN pre-treatment was shown to be able to prevent membrane damage, DNA fragmentation, and ROS accumulation [17]. A previous study evidenced that SFN was able to exert concentration-dependent protection to DNA damage caused by exposure to low levels of pesticide in both the pre-incubation and co-incubation strategies [18]. In an in vivo PD 6-OHDA-induced model, SFN administration ameliorated motor coordination and counteracted apoptosis through the inhibition of DNA fragmentation and caspase-3 activation [15].

The link between DNA damage and neurodegeneration is already well established. In PD, DNA damage accumulation occurs before PD onset, indicating its direct involvement in the PD pathophysiology. Interestingly, the loss of function of some DNA repair proteins in mice recapitulated some features of the disease phenotype. Moreover, some proteins playing a role in PD are directly involved in DNA repair. This evidence suggested that DNA damage may represent a direct mechanism of neurodegeneration in PD. Moreover, 8-OHdG and γ-H2AX, a marker of oxidative DNA damage and a DNA double-strand breaks (DSBs) marker, respectively, increased in PD [19]. Elevated levels of DSBs were detected in the dopaminergic neurons of the substantia nigra in PD patients and in mouse models of PD. Moreover, DSBs accumulation preceded the onset of motor phenotype and dopaminergic degeneration and is correlated to PD severity [20].

In this study, we observed that the pre-treatment with GRA + MYR modulated DEGs involved in oxidative stress and DNA repair, as demonstrated by both GO, Reactome, and network analysis.

GO analysis showed the overrepresentation of BP related to oxidative stress response, while Reactome analysis evidenced the overrepresentation of pathways involved in DNA repair and, particularly, DSB repair.

MCL network analysis evidenced clusters whose DEGs were involved in DNA processes, including DNA repair and replication. Also, k-means analysis network analysis revealed that the major cluster was “DNA Double-Strand Break Repair.” Network functional analysis of the 62 DEGs belonging to the “DNA Double-Strand Break Repair” cluster revealed a complex and coordinated modulation of pathways involved in the genomic damage response following pre-treatment with GRA + MYR prior to MPP^+^ exposure. Overall, the transcriptomic profile suggested a modulation of DEGs involved in the DNA damage response, oriented toward the maintenance of cellular homeostasis and chromatin stability.

Among the significantly upregulated genes in the PRE-TREAT group, *RAD51* represents a key component of homologous recombination repair [21], indicating a potential enhancement of high-fidelity mechanisms responsible for the resolution of DSBs. In parallel, the increased expression of *POLD3* and *CHTF18* in the PRE-TREAT group, both involved in replication fork progression and genomic stability [22,23], supports the hypothesis of preserved replicative integrity under neurotoxic stress conditions.

Conversely, several central genes involved in the DNA damage response were moderately downregulated in the PRE-TREAT group, including *ATM* [24], *PRKDC* [25], *TP53BP1*, *UIMC1*, *WRN*, and *EXO1*. This pattern may reflect a reduced requirement of proteins involved in DNA damage sensing and signaling pathways, suggesting that GRA + MYR pre-treatment may limit the extent of MPP^+^-induced damage, thereby attenuating the recruitment of DNA damage response complexes typically associated with severe or persistent genomic injury.

Moreover, persistent activation of ATM signaling has been demonstrated in mouse models of both PD and other neurodegenerative diseases, while targeted inhibition of ATM activity has neuroprotective effects [19]. Targeting the DNA damage response via ATM inhibition reduced genotoxic stress, suppressed neuroinflammation, and improved behavioral outcomes in a mouse model of α-synucleinopathy [26].

*ATM*, together with *TP53BP1* and *RPA1*, represented the three DEGs with higher betweenness score in the network. RPA1 is a main regulator of different DNA metabolic processes, being involved in replication, recombination, and repair [27], while TP53BP1 has a role in the maintenance of genome integrity, being involved in DSB repair [28]. All of them were downregulated by bioactivated GRA pre-treatment, suggesting that GRA + MYR may reduce the need for DNA repair machine in the PD model.

The modulation of epigenetic and chromatin remodeling genes appeared particularly relevant. Indeed, *KDM1A*, *MBTD1*, *SGF29*, *SMARCA4*, *SMARCB1*, and *SUPT16H* were downregulated in the PRE-TREAT group, whereas several histone-related genes, including *H4C1*, *H2BC11*, *H3C10*, *H2BC4*, and *H2BC9*, showed increased transcriptional expression in the PRE-TREAT group. This expression pattern suggested a dynamic chromatin reorganization potentially associated with enhanced nucleosomal plasticity and fine regulation of DNA accessibility during the oxidative stress response.

It is also noteworthy that genes involved in proteostasis and cellular survival, such as *BECN1*, *STUB1*, *VCP*, and *HSPA2*, were concomitantly upregulated in the PRE-TREAT group, suggesting a coordinated modulation of autophagy, proteostasis, and protein quality control pathways. This response may contribute to reduce the accumulation of damaged proteins and intracellular oxidative burden.

The increased expression of *NFKBIA* by GRA + MYR pre-treatment, an inhibitor of the NF-κB pathway, further suggests a potential attenuation of the inflammatory and stress-related response induced by MPP^+^. Similarly, the upregulation of *TFRC*, *FTH1*, and *FTL* in the PRE-TREAT group indicated a modulation of iron metabolism, a process tightly linked to oxidative stress and dopaminergic neurodegeneration.

Overall, these findings suggested that GRA + MYR pre-treatment may exert a cytoprotective effect through the selective modulation of DEGs involved in DNA DSB repair pathways, suggesting the promotion of mechanisms involved in the maintenance of genomic integrity while reducing the need for DNA damage-dependent responses. This transcriptomic profile appears consistent with a potential neuroprotective effect against MPP^+^-induced toxicity.

The post-treatment with bioactivated GRA appeared to induce a various transcriptomic profile. GO analysis of the post-treatment with bioactivated GRA evidenced the overrepresentation of processes related to the response to oxidative stress and detoxification. These results were in line with those of Reactome pathway analysis. Interestingly, genes associated with antioxidant responses, such as those involved in glutathione pathways (*GCLC* and *GCLM*) and antioxidant enzymes (*SOD1* and *NQO1*), were upregulated in POST-TREAT compared to MPP^+^_post_. Western blot analysis confirmed transcriptomic data, highlighting the increase in SOD1 protein levels.

MCL cluster analysis evidenced a major cluster related to chromatin organization. K-means network analysis needed a secondary clustering step to obtain a specific characterization. The sub-clusters that were identified were “Chromatin organization,” “Regulation of transcription elongation from RNA polymerase II promoter/Integrator complex,” “Nicotinate and nicotinamide metabolism,” and “Antigen processing and presentation/Nef-mediated down-modulation of cell surface receptors.” The nodes with highest betweenness of the complete network were *RPS27A* and *TP53*. RPS27A, whose expression was upregulated in the POST-TREAT group, assembled into ribosomes, but it plays also roles independent of ribosomes. It is also involved in DNA repair and p53 pathway [29]. Then, it is also linked with TP53, which also plays a role in dopaminergic neuron cell death in neurotoxic conditions [30]. Interestingly, *TP53* is downregulated by GRA + MYR post-treatment.

Then, we looked at the DEGs that connected the different subnetworks. Interestingly, “Chromatin organization” and “Antigen processing and presentation/Nef-mediated down-modulation of cell surface receptors” were connected by *HSPA5* and *PDIA3*, belonging to these subnetworks, respectively. Both proteins play a role in ER functions and stress. Specifically, PDIA3 supports proper protein folding, while HSPA5 represents a regulator of ER homeostasis and the unfolded protein response (UPR). *PDIA3* and *HSPA5* were, respectively, upregulated and downregulated in POST-TREAT group. In addition, a previous study in a 6-OHDA rat model of PD found decreased levels of ERp57, encoded by *PDIA3* in the midbrain, suggesting that reduction of the components of quality control system may play a role in neuronal injury in PD [31]. Then, these results suggested that GRA + MYR may support quality control system, avoiding the activation of UPR.

The DEGs *HK2*, *G6PC3*, *G6PD*, and *RPL37A* of the subnetwork “Chromatin organization” were connected to the DEGs *ALDOC*, *PKM*, *TKT*, and *LDHA* of the subnetwork “Nicotinate and nicotinamide metabolism.” These DEGs evidenced that the treatment with GRA-MYR may induce a metabolic reprogramming. Indeed, neurons can use glucose metabolism for antioxidant purposes at the expense of its role in bioenergetics, thus downregulating glycolysis [32]. The DEGs involved in glycolysis *ALDOC*, *PKM*, *HK2*, and *LDHA* were downregulated in the POST-TREAT group, in association with the upregulation of *G6PD* and *TKT*, suggesting a glucose shift from glycolysis to pentose phosphate pathway (PPP). As described in literature, the increase in G6PD associates to PPP activation in neurodegenerative, and PD experimental models represent a protective mechanism because they provide the cells with NADPH, crucial to counteract oxidative stress as substrate for glutathione reductase [33,34].

The DEGs *THRA*, *HDAC1*, *H4C9*, *H3C13*, *H2BC3*, *H4C2*, *H2AC13*, *H4C8*, *H2AC15*, *H2AC16*, *H2AC8*, and *EHMT2* linked the subnetwork “Chromatin remodelling” with “Regulation of transcription elongation from RNA polymerase II promoter/Integrator complex” through the DEGs *MED14*, *H2AZ1*, *MED25*, *SUPT6H*, *POLR2E*, *POLR2L*, and *LEO1*. These DEGs evidenced a modulation of chromatin remodelling and RNA transcriptional mechanism. Indeed, transcription was shown to be altered in PD models [35]. Interestingly, in the POST-TREAT group, we found the downregulation of *HDAC1* and *EHMT2*, and their inhibition was shown to alleviate experimental PD [36,37]. Moreover, various histones were modulated by GRA + MYR treatment, indicating a structural remodelling of chromatin. Interestingly, the gene *H2AZ1* was found upregulated in the POST-TREAT group. Ablation of H2A.Z in various neuronal subtypes triggers cell death. It was shown that H2A.Z exerted a neuronal survival effect, binding to the promoters of key nuclear-encoded mitochondrial genes to regulate their expression and promote organelle function [38].

However, a limitation of this exploratory study is that RNA-seq experiments were performed with only two biological replicates per condition. While the data provides valuable insights into GRA-MYR neuroprotective effects in PD, a validation on a larger sample size will be beneficial. Moreover, the results related to DNA damage repair need to be validated.

Altogether, these results suggested that GRA + MYR can exert protective effects: the pre-treatment can modulate DEGs involved in DNA damage repair. In post-treatment, DNA processes seemed to be modulated in association with an antioxidant effect that may counteract MPP^+^-induced cytotoxicity.

## 4. Materials and Methods

### 4.1. Plant Material and GRA and MYR Extraction

GRA was extracted from Tuscan black kale (*Brassica oleracea*) seeds, while the extract rich in MYR was obtained by *Sinapis alba* seeds using hydrodynamic cavitation by Hyres. In liquid media, cavitation represents a complex multiphase process characterized by the formation, expansion, and near-adiabatic collapse of vapor bubbles within a fluctuating pressure field. The violent collapse induces severe pressure shockwaves, high-velocity liquid microjets, localized extreme temperatures, and free radical production, in particular, hydroxyl radicals [39,40]. Hydrodynamic cavitation is distinguished from other cavitation technologies by its industrial scalability. Generated by passing liquids or liquid–solid mixture through static constrictions or via submerged rotary devices, hydrodynamic cavitation methods are highly efficient across diverse uses, including food processing and natural product extraction [41,42,43].

APTOSol performed the drying and isolation of the dry extract via spray-drying techniques.

Different batches underwent comprehensive analytical characterization to evaluate their GLs composition and identify the extract most suitable for the following activities. Analyses were performed using the UHPLC-ESI-MS/MS method. In addition to GRA, glucoerucin and progoitrin were also quantified, allowing for a comprehensive quality assessment of the resulting extracts.

The results identified the batch characterized by significantly higher concentrations of the main GLs. Notably, the average GRA content was 170.67 mg/g, confirming the effectiveness of the extraction, purification, and drying conditions adopted during the final phases of process development. The content of glucoerucin and progoitrin was 39.00 mg/g and 1.33 mg/g, respectively.

The extracts were treated with gamma radiation for microbial decontamination. This process was required because the same extracts are also intended for administration in a clinical trial (NCT07360977).

**Figure 15:**
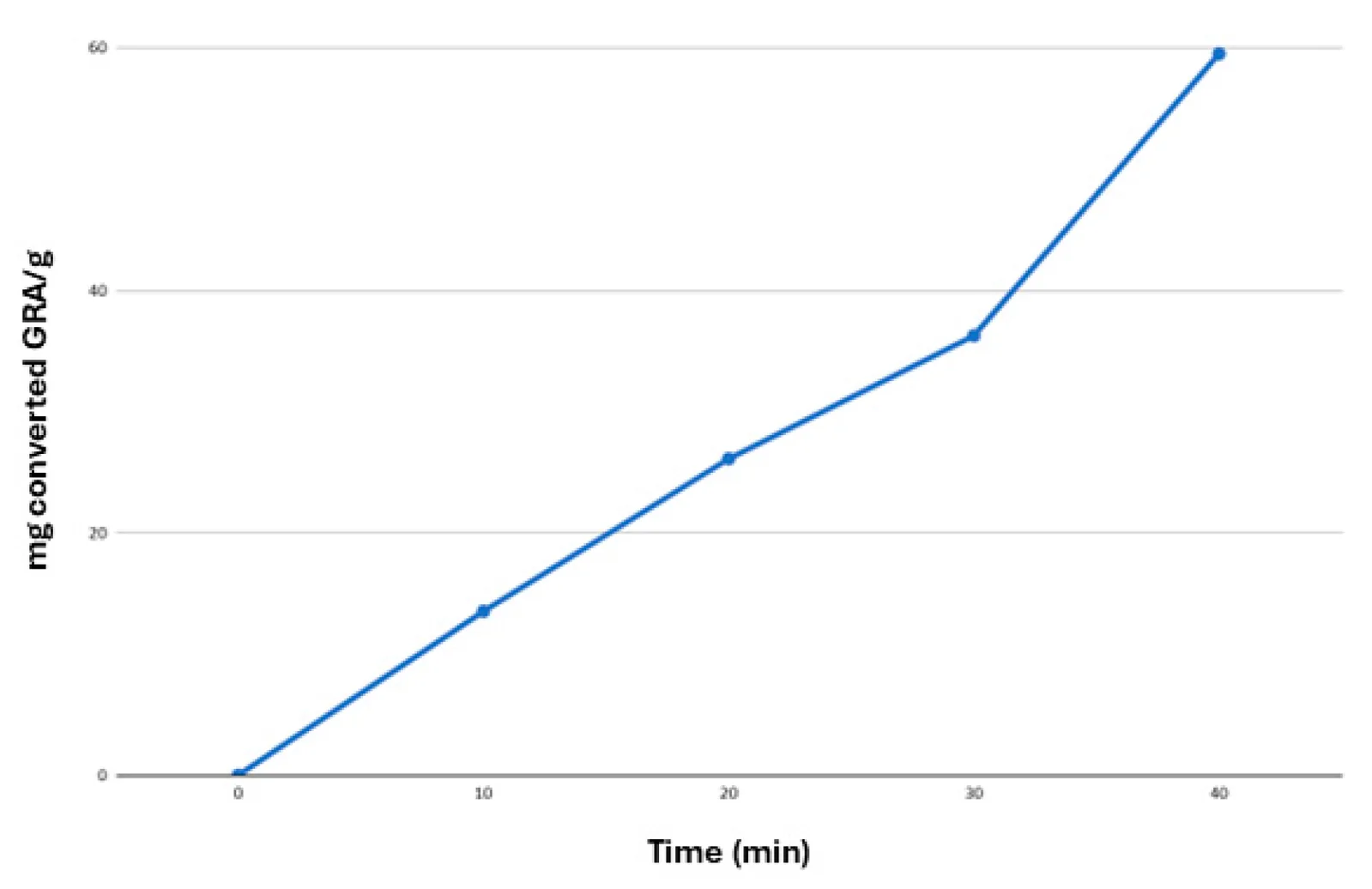
MYR activity evaluation. The results demonstrated a progressive conversion of GRA during incubation.

To quantitatively evaluate the activity of the MYR, a dedicated kinetic assay was established via UHPLC-ESI-MS/MS. The results demonstrated a progressive conversion of GRA during incubation, confirming the retention of enzymatic activity in the MYR preparation (Figure 15).

### 4.2. Cell Culture and Treatment

The SH-SY5Y cell line, purchased from the American Type Culture Collection (ATCC) (Manassas, VA, USA), was maintained in Dulbecco’s Modified Eagle’s Medium/Nutrient Mixture F-12 Ham (DMEM/F12) medium (Sigma-Aldrich, Saint Louis, MO, USA), containing 10% fetal bovine serum (FBS) (Sigma-Aldrich, Saint Louis, MO, USA), 1% glutamine, and 1% penicillin-streptomycin. With the aim of inducing neuronal differentiation, SH-SY5Y cells were incubated with 10 µM retinoic acid (RA) for 5 days. In order to bioactivate GRA, both GRA and MYR were dissolved in PBS and were incubated at 45 °C for 45 min to induce the hydrolysis of GRA to form its ITC, SFN. At the end of the 5-day differentiation, in the PRE-TREAT group, the medium was replaced with fresh medium with or without GRA + MYR at a concentration of 5 µM dissolved in PBS. After 24 h of pre-treatment, the medium was changed with fresh medium with 1 mM MPP^+^ dissolved in water for 48 h. In the POST-TREAT group, differentiated cells were incubated for 48 h with 1 mM MPP^+^. After 48 h, the medium was replaced with fresh medium with or without bioactivated GRA, at a concentration of 5 µM for 24 h. Bioactivated GRA concentration was chosen on the basis of a previous study in which bioactivated GRA was not toxic in the range 1–5 µM in differentiated SH-SY5Y cells, and it was able to exert neuroprotective effects [44]. MPP^+^ concentration and time of exposure were chosen based on a previous report [45]. In this previous study, a time and dose-dependent decrease of cell viability in RA-differentiated SH-SY5Y treated with MPP^+^ was observed. After 24 h, the loss of cell viability was low, while after 48 h, all the tested concentrations (range 0.5–2 mM) caused a significant reduction of cell viability. We chose to perform experiments using 1 mM for 48 h because a reduction of 30/40% of cell viability indicates a sublethal condition, commonly used for studies of cytotoxicity and neuroprotection. Indeed, 60–70% viability represents an optimal window to study molecular mechanisms without overwhelming the culture with massive cell lysis. Control cells were incubated with DMEM/F12 medium supplemented with 10% FBS.

In summary, the specific groups used in this study are

- MPP^+^_pre_: at the end of the differentiation with RA, cells were incubated for 24 h with cell culture medium and then treated for 48 h with 1 mM MPP^+^.
- PRE-TREAT: at the end of the differentiation with RA, cells were exposed for 24 h to 5 µM GRA + MYR and then treated for 48 h with 1 mM MPP^+^.
- MPP^+^_post_: at the end of the differentiation with RA, cells were treated for 48 h with 1 mM MPP^+^; then cells were incubated for 24 h with cell culture medium.
- POST-TREAT: at the end of the differentiation with RA, cells were treated for 48 h with 1 mM MPP^+^; then cells were exposed for 24 h to 5 µM GRA + MYR.

### 4.3. MTT Assay

In order to evaluate cell viability, SH-SY5Y cells were cultured in 96-well plates, treated as reported in the previous paragraph, and MTT assay was carried out. At the end of the treatment, cells underwent medium replacement with Thiazol Blue Tetrazolinium Bromide (MTT) at a concentration of 0.5 mg/mL (Sigma-Aldrich Merck KGaA, Darmstadt, Germany) for 4 h at 37 °C. The formed crystals were dissolved using acidic isopropanol. The optic density was measured spectrophotometrically using microplate reader BioTek Synergy H1 microplate reader. Experiments were carried out in triplicate.

### 4.4. Transcriptomic Analyses and Bioinformatics Processing

At the end of the treatments, cells were harvested, and Maxwell^®^ RSC simplyRNA Cells Kit (Promega, Madison, WI, USA) was used to extract RNA (RIN > 9), following the manufacturer’s instructions. TruSeq RNA Exome protocol (Illumina, San Diego, CA, USA) was used for the library preparation. Experiments were carried out in duplicate. The library was analyzed using the Illumina instrument NextSeq 550 Dx.

Initial quality control of raw reads was assessed using FastQC v0.11.9, followed by adapter removal and quality trimming with Trimmomatic v0.40-rc1 [46]. Cleaned reads were aligned to the human reference genome (GENCODE hg38 v39) using STAR RNA-seq aligner v2.7.10a_alpha_220207 [47]. Quality and mapping information are included in Supplementary Table S11. Gene counts were obtained using featureCounts v2.0.1 [48] and processed for differential expression analysis using the DESeq2 library v1.36.0 [49] in R v4.2.0. *p*-values were adjusted using the Benjamini–Hochberg procedure, with a significance cutoff set at q-value < 0.05. In comparison MPP^+^_pre_ vs. PRE-TREAT, the FC level was computed as $\log_{2}\left(\frac{PRE-TREAT}{MPP+pre}\right)$. In the comparison MPP^+^_post_ vs. POST-TREAT, the FC level was computed as $\log_{2}\left(\frac{POST-TREAT}{MPP+post}\right)$. Finally, GO overrepresentation analysis of the identified DEGs was conducted using clusterProfiler v4.4.3, using, as background, the genes tested in DESeq2 [50]. DEG interaction networks and pathway enrichments were further explored using the STRING [51] (accessed on 21 June 2026) and Reactome databases [52] (accessed on 18 June 2026), respectively.

To explore the biological interaction networks among DEGs, protein–protein interaction (PPI) networks were constructed using the STRING database (version 12.0) with a high-confidence interaction score threshold (>0.7), retaining experimental and database sources. To dissect the modular architecture of the resulting networks without introducing a priori bias regarding cluster numbers, we employed the MCL with an inflation parameter set to 3.0, as implemented in STRING by default. For comparative purposes and global topological assessment, k-means clustering based on the number of disconnected components was also performed. While k-means provided a broad macro-level organization of the network components, MCL allowed the identification of tightly connected, fine-grained functional modules based on network topology and flow dynamics.

### 4.5. Protein Extraction and Western Blot

At the end of the treatments, cells were harvested, and protein extraction was carried out using RIPA (Thermo Scientific™, Waltham, MA, USA), according to the manufacturer’s instructions. Bradford assay (Bio-Rad, Hercules, CA, USA) was used in order to evaluate protein concentrations. In total, 20 micrograms of proteins were heated for 5 min at 95 °C and then loaded and resolved using SDS-polyacrylamide gel electrophoresis (SDS-PAGE). Proteins were transferred onto a PVDF membrane (Immobilon-P, Millipore, Burlington, MA, USA). After blocking with 5% skim milk dissolved in TBS for 1 h at room temperature, membranes were incubated overnight at 4 °C with the antibody: SOD1 (1:1250; Sigma-Aldrich, Saint Louis, MO, USA). Then, membranes were washed with TBS 1× and incubated with HRP-conjugated anti-rabbit (1:2000; Santa Cruz Biotechnology Inc., Dallas, TX, USA) for 1 h at room temperature. The protein bands were visualized using the Immobilion Forte Western HRP Substrate (Millipore Corporation, Burlington, MA, USA), and their acquisition was obtained using the ChemiDoc™ XRS + System (Bio-Rad, Hercules, CA, USA). Protein band quantification was performed using ImageJ 1.53t software. Membranes were stripped using Restore Western Blot buffer (Thermo Scientific, Meridian, Rockford, IL, USA), and after, they were incubated with GAPDH HRP-conjugated antibody (1:1000; Cell Signaling Technology, Danvers, MA, USA), used as loading controls. The original uncropped blots are available in Supplementary Figure S3.

### 4.6. Statistical Analysis for MTT Assay

Statistical analysis for cell viability and Western blot was performed using GraphPad Prism version 7.0 software (GraphPad Software, La Jolla, CA, USA). One-way ANOVA test followed by the Bonferroni post hoc test was applied for multiple comparison for cell viability. The *t* test was used for the comparison between two groups in Western blot. A *p*-value ≤ 0.05 was considered statistically significant. The results are expressed by mean ± standard deviation (SD).

## 5. Conclusions

GRA + MYR showed to be able to exert neuroprotective effects in an in vitro model of PD, both as pre- and post-treatment, counteracting MPP^+^ toxicity. Our results evidenced that the pre-treatment modulated DEGs involved in DNA-related processes, suggesting a potential protective response against DNA damage induced by MPP^+^. Moreover, the post-treatment was able to induce a transcriptomic profile associated with an antioxidant action, as demonstrated also by SOD1 increase. We can suppose that GRA + MYR exerts a potential protective action, modulating DEGs involved in DNA repair, but it may also be able to exert an antioxidant response against MPP^+^. These transcriptomic results are exploratory and need to be replicated and validated in larger sample sizes, especially for DEGs involved in DNA repair.

## Figures and Tables

**Table 1:** Overrepresented BP related to oxidative stress and DNA processes in the comparison MPP^+^_pre_ vs. PRE-TREAT with the included DEGs with log_2_ FC threshold of ≥2 or ≤−2.

ID	Description	q Value	Gene
GO:1901298	Regulation of hydrogen peroxide-mediated programmed cell death	8.74 × 10^−3^	*HGF*
GO:0010421	Hydrogen peroxide-mediated programmed cell death	8.74 × 10^−3^	
GO:0097468	Programmed cell death in response to reactive oxygen species	8.74 × 10^−3^	
GO:1903206	Negative regulation of hydrogen peroxide-induced cell death	8.74 × 10^−3^	
GO:1901032	Negative regulation of response to reactive oxygen species	8.74 × 10^−3^	
GO:1902883	Negative regulation of response to oxidative stress	9.43 × 10^−3^	
GO:1903205	Regulation of hydrogen peroxide-induced cell death	9.74 × 10^−3^	
GO:0036474	Cell death in response to hydrogen peroxide	1.00 × 10^−2^	
GO:1901031	Regulation of response to reactive oxygen species	1.02 × 10^−2^	
GO:1903202	Negative regulation of oxidative stress-induced cell death	1.18 × 10^−2^	
GO:1903201	Regulation of oxidative stress-induced cell death	1.35 × 10^−2^	
GO:2000573	Positive regulation of DNA biosynthetic process	1.40 × 10^−2^	
GO:1900407	Regulation of cellular response to oxidative stress	1.42 × 10^−2^	
GO:0070301	Cellular response to hydrogen peroxide	1.45 × 10^−2^	
GO:1902882	Regulation of response to oxidative stress	1.50 × 10^−2^	
GO:0036473	Cell death in response to oxidative stress	1.50 × 10^−2^	
GO:0046322	Negative regulation of fatty acid oxidation	8.74 × 10^−3^	*SOX9*
GO:0046320	Regulation of fatty acid oxidation	1.01 × 10^−2^	

**Table 2:** Major MCL network functional cluster.

Cellular Responses	Clusters	Genes
Signal transduction	Heterotrimeric G-protein complex and regulator of G protein signalling domain	*MMP15*, *GNAI2*, *GNAI1*, *RGS9*, *RGS5*, *CXCR4*, *RIC8A*
DNA double-strand break repair	Impaired BRCA2 binding to RAD51	*RPA1*, *RAD51*, *WRN*, *EXO1*
DNA replication-dependent/independent chromatin reorganization	DNA replication-independent chromatin organization Structural constituent of chromatin Deposition of new CENPA-containing nucleosomes at the centromere:	*SUPT16H*, *H2BC4*, *H4C1*, *H4C13*, *CENPH*, *H2BC9*
	Bromodomain CENP-A containing chromatin and DNA replication-dependent chromatin assembly	*H3C10*, *H2BC11*, *BPTF*, *SMARCB1*, *GLYR1*, *TRIM24*

**Table 3:** Overrepresented BP in the comparison MPP^+^_post_ vs. POST-TREAT with the included DEGs with log_2_ FC higher than 2 or lower than −2.

ID	Description	q Value	Gene
GO:0019430	Removal of superoxide radicals	3.14 × 10^−3^	*H19*, *NQO1*
GO:0071450	Cellular response to oxygen radical	3.14 × 10^−3^	*H19/NQO1*
GO:0071451	Cellular response to superoxide	3.14 × 10^−3^	*H19*, *NQO1*
GO:0000303	Response to superoxide	3.14 × 10^−3^	*H19*, *NQO1*
GO:0000305	Response to oxygen radical	3.14 × 10^−3^	*H19*, *NQO1*
GO:0006801	Superoxide metabolic process	1.29 × 10^−2^	*H19*, *NQO1*
GO:0098869	Cellular oxidant detoxification	2.27 × 10^−2^	*H19*, *NQO1*
GO:0062014	Negative regulation of small-molecule metabolic process	2.30 × 10^−2^	*ERFE/H19*
GO:1990748	Cellular detoxification	2.30 × 10^−2^	*H19*, *NQO1*
GO:0097237	Cellular response to toxic substance	2.43 × 10^−2^	*H19*, *NQO1*
GO:0098754	Detoxification	2.99 × 10^−2^	*H19*, *NQO1*

## Data Availability

The data supporting the findings of this study are openly available in the NCBI Sequence Read Archive at BioProject, accession number PRJNA1517608.

## Supplementary Materials

The following supporting information can be downloaded at https://www.mdpi.com/article/10.3390/ijms27188232/s1.

## Abbreviations

The following abbreviations are used in this manuscript:

PD	Parkinson’s disease
ROS	Reactive oxygen species
GLs	Glucosinolates
ITC	Isothiocyanate
MYR	Myrosinase
GRA	Glucoraphanin
SFN	Sulforaphane
BBB	Blood–brain barrier
GRA + MYR	GRA bioactivated with MYR
MPP^+^	1-methyl-4-phenylpyridinium
PRE-TREAT	Pre-treatment with GRA + MYR
POST-TREAT	Post-treatment with GRA + MYR
DEGs FC	Differentially expressed genes Fold change
GO	Gene Ontology
BP	Biological Process
HO-1	Heme oxygenase-1
DSBs	Double-strand breaks
UPR	Unfolded protein response
PPP	Pentose phosphate pathway
FBS	Fetal bovine serum
RA	Retinoic acid
MTT	Thiazol Blue Tetrazolinium Bromide
