# Supplementary material for: Bioactivated Glucoraphanin Exerts a Potential Protective Effect in an In Vitro Model of Parkinson’s Disease: A Transcriptomic Study
Source: Int J Mol Sci. 2026 Sep 16;27(18):8232. doi: 10.3390/ijms27188232 (PMC13607179; doi:10.3390/ijms27188232)
Supplement: Supplementary file 1 [file ijms-27-08232-s001.zip › Table S11.pdf]

Table S11. Quality information and mapping related to the different samples used in the comparative transcriptomics analysis.

Sample	Depth	# Input Reads	Mapping %
MPP^{+}_{pre}-1	89.89	15594122	76.05%
MPP^{+}_{pre}-2	75.38	13425719	72.34%
MPP^{+}_{post}-1	69.87	13057734	69.37%
MPP^{+}_{post}-2	51.90	10677690	64.56%
PRE-TREAT-1	64.57	11243072	71.99%
PRE-TREAT-2	77.44	13743341	73.10%
POST-TREAT-1	77.19	14503030	71.04%
POST-TREAT-2	91.69	16254967	76.04%
